# Identifying genetic susceptibility loci associated with human coronary artery disease

**DOI:** 10.1371/journal.pone.0315460

**Published:** 2025-01-09

**Authors:** Aqsa Zahid, Andleeb Batool, Abdul Wajid, Yurong Wu, Chun Liang, Muhammad Ajmal Khan, Amin Ullah, Kashif Iqbal Sahibzada, Hong Xue

**Affiliations:** 1 Department of Zoology, GC University, Lahore, Pakistan; 2 Baluchistan Universities of Information Technology, Engineering and Management Science, Quetta, Pakistan; 3 Division of Life Science, Hong Kong University of Science and Technology, Clear Water Bay, Hong Kong, China; 4 Department of Allied Health Sciences, Iqra National University Peshawar, Peshawar, Pakistan; 5 University of Lahore, Lahore, Pakistan; UCMI: University College MAIWP International, MALAYSIA

## Abstract

Coronary artery disease (CAD) is a multigenic condition influenced by both nature and nurture (60% to 40%). Prognosis of CAD is based on familial patterns. This study examined and analyzed the susceptibility of CAD to genetic variants in various Pakistani families. A total of 50 families, 308 participants (79 affected and 229 unaffected were genotyped for *NOS3* (rs1799983, rs2070744), *PON1* (rs662), *LPA-PLA2* (rs105193, rs1805017), *APOE* (rs429358, rs7412), *PCSK9* (rs505151), *MEF2A* (rs325400), *TNF* (rs1800629) and *LDLR* (rs1122608, rs2228671) genes. The family-based association in CAD associated genes SNPs were *NOS3* (rs1799983), *PON1* (rs662), *LPA-PLA2* (rs1805017), *MEF2A* (rs325400), and *LDLR* (rs1122608, rs222867) showed transmission within families p≤ 0.05 whereas *NOS3* (rs2070744), *APOE* (rs429358, rs7412) and *TNF* (rs1800629) showed no association TDT asymptotic p-value >0.05. In DFAM and QFAM test *NOS3* (rs1799983), *PON1* (rs662), *MEF2A* (rs325400), and *LDLR* (rs1122608, rs222867) showed positive association p≤ 0.05 in both whereas *NOS3* (rs2070744), *APOE* (rs429358, rs7412), *LPA-PLA2* (rs1805017) and *TNF* (rs1800629) showed low risk of transmission asymptotic p-value >0.05 in DFAM but *NOS3*(rs2070744), *APOE*(rs7412), *LPA-PLAG2*(rs1805017) also showed association p≤ 0.05 whereas *APOE* (rs429358) and *TNF* (rs1800629) showed no association EMP1 *p*-value >0.05 in QFAM. In linkage analysis Chromosome 6 (Position 70.810): **LOD = 3.16**, Chromosome 7 (Position 107.190): **LOD = 3.16**, and chromosome 19 (Position 31.470): **LOD = 3.90** also showed significant association with disease as p < 0.05. This discovery enhances the understanding about genetic variants of CAD and also facilitates early detection, targeted interventions, pattern of inheritance in population. This ultimately improving patient outcomes and guiding future research to highlight its significance as a potential diagnostic marker.

## Introduction

Coronary artery disease (CAD) is a leading cause of death worldwide and continues to be a significant public health issue. This highlights the importance of identifying molecular markers to detect individuals at high risk of developing CAD [1]. CAD is one of the primary cardiac diseases affecting the global population. The prevalence of this disease is expected to increase among healthy individuals due to comorbidities, lifestyle factors, environmental influences, and genetic variability [2]. In developed countries, CAD is the leading cause of death, mainly resulting from the thickening and hardening of the coronary arteries. The prevalence of CAD is associated with factors such as age, gender, lifestyle, family history, and economic status [3]. Various genetic factors significantly contribute to the risk of CAD, and substantial progress has been made in this area over the past decade [4]. Low- and middle-income countries, including those in South Asia—such as Afghanistan, Bangladesh, Bhutan, India, Maldives, Nepal, Pakistan, and Sri Lanka—account for Ethnic factors, hypertension, dyslipidemia, abdominal obesity may increase their susceptibility to coronary artery disease (CAD) [5]. Epidemiological data indicates that South Asians are almost twice as likely as Europeans and five times as likely as Chinese to develop CAD [6]. With a population of 1.86 billion, or about 24% of the global population, South Asia—which includes India, Pakistan, and Sri Lanka—has a notably higher prevalence of CVD and type 2 diabetes mellitus (T2DM) compared to Western countries [7]. A significant missense SNP, rs2075291 in the *APOA5* gene, has been associated with CAD at a genome-wide significance level in diverse Southeast Asian populations [8]. In North Indian populations, seven gene polymorphisms have shown independent associations with increased CAD risk, with *AGT*, *APOA5/C3* haplotypes, and elevated genetic risk scores demonstrating substantial risk, which has potential implications for both clinical and public health strategies [9]. Lipoproteins play a vigorous role in the prognosis of CAD. So, with their susceptibility to affect lipid levels, the concurrence of risk factors of the CAD with *APOE*, *LDLR* and *APOB* polymorphisms maybe be one of the causes in development of CAD [10]. The *MEF2* protein family is closely linked with Ca^2+^ signaling was earlier supposed to one of the main cause in the development of heart and muscle but Modern research showed that they are also related with development and prognosis of many other diseases [11]. *MEF2A* gene, specifically the rs325400, could be considered a predisposition element for CAD. The allele T increases the risk of CAD amid Saudis. However, other variants detected in this gene show no association with CAD in the Saudi population [12]. *PON1* is a plasma linked enzyme to a higher possibility of coronary artery disease (CAD) that can slow the development of atherosclerosis. The expression of protein of *PON1* was seen in human tissue(aortic) and is crucial to the development of CAD [13]. The Q192R polymorphism was found to increase the jeopardy of CAD across several genetic representations. In one of the model, the odds ratio was 1.35 with a confidence interval (CI) of 1.02–1.79. In the allelic model, the OR was 1.16 with a CI of 1.00–1.33, and in the dominant model, the OR was 1.25 with a CI of 1.03–1.52 [14]. Apolipoprotein E *(ApoE)* is an important factor of various lipoproteins and plays a noteworthy role in cholesterol and lipid transport amid cells in different tissues. These alleles code for the isoforms E2/ E3 and E4 which have different binding affinities for their corresponding receptors [15]. TNF-α polymorphisms are great influence in the prognosis and development of the CAD [16]. TNF-αlpha is a cytokine pro-inflammatory that may play a crucial role in the pathogenesis of CAD. Patients with the A/A genotype at *TNF-α* (−308) exhibit significantly higher *TNF-α* mRNA expression levels compared to healthy individuals [17]. In developed countries, the death rate from CAD has worsened since 1980. Meanwhile, developing and underdeveloped countries bear 75% of the over-all CAD burden. The South Asian countries are on a significantly higher danger, with a prevalence 50% to 300% greater than the allover of the world [18]. *ENOS* produced by oxidation of l-arginine to l-citruline for the action at the) is deliberated an imperative CAD protective factor [19]. The *PCSK9* comprises of 12 exons that encode a 692 amino acid glycoprotein was the third related with hypocholesteremia providing evidence in progression of CAD. However, different researches have shown controversial results regarding whether PCSK9 variations are risk factors for CAD [20]. Lipoprotein-associated phospholipase A2 *(Lp-PLA2)* is located at 6p21.2 comprises on 12 exons an enzyme involved in both lipoprotein metabolism and inflammatory pathways produced such compounds that are responsible in progression of atherogenesis, making it a potential therapeutic target for coronary heart disease (CHD) [21]. These findings suggest that polymorphisms in various genes may contribute in the progression as well as development of CAD. As molecular genetics advances, it becomes increasingly important to study the genetic transmission of CAD in large populations, including our own and others. Polymorphisms in genes such as *NOS3 (rs1799983*, *rs2070744)*, *PON1 (rs662)*, *LPA-PLA2 (rs105193*, *rs1805017)*, *APOE (rs429358*, *rs7412)*, *PCSK9 (rs505151)*, *MEF2A (rs325400)*, *and LDLR (rs1122608*, *rs2228671)* related to CAD have been studied. Previous studies on different gene variants and their polymorphisms in the Pakistani population have primarily been case-control studies. But our research is unique as it is the first study focusing on *NOS3 (rs1799983*, *rs2070744)*, *PON1 (rs662)*, *LPA-PLA2 (rs105193*, *rs1805017)*, *APOE (rs429358*, *rs7412)*, *PCSK9 (rs505151)*, *MEF2A (rs325400)*, *and LDLR (rs1122608*, *rs2228671)* to investigate different genetic variants, pattern of inheritance and their relation with CAD specifically in families in Pakistan.

Therefore, our aim is to explore the association of *NOS3 (rs1799983*, *rs2070744)*, *PON1 (rs662)*, *LPA-PLA2 (rs105193*, *rs1805017)*, *APOE (rs429358*, *rs7412)*, *PCSK9 (rs505151)*, *MEF2A (rs325400)*, *and LDLR (rs1122608*, *rs2228671)* in families affected by CAD within the Pakistani population. Due to ethnicity and narrow gene pool this study is also important for identifying how these selected genetic variations are inherited among families. The identification of specific genetic variants linked to CAD among Pakistani families reinforces the role of hereditary factors in the disease’s prevalence within this population. This discovery underscores the importance of studying to uncover population-specific strategy of risk assessment, pattern of inheritance, screening of high risk individual within families and open avenue for personalized medicine.

## Materials and methods

### Ethics approval of the study

The study was approved by the Ethical Committee of the Institute of Punjab cardiology and Board of Advance Research, department of Zoology, G.C.U. Lahore and. The study was reviewed ethically and approved by the Punjab Institute of Cardiology' s ethical review committee (Ref No: RTPGME-Research-107) and Government College University Lahore' s Institutional Bioethics Committee (GCU-IIB-956).

### Diagnostic criteria of patients

The patients included in the study were clinically diagnosed with CAD by physicians in accordance with World Health Organization (WHO) guidelines. Diagnosing of CAD depends on a combination of medical history assessment, physical examination, patient’s lifestyle, positive family history. incidence of medical symptoms, such as chest tightness, and blood profiling (incidence of cholesterol, hypertension, lipid profile etc.), echocardiogram(ECG) and coronary angiography.

### Inclusion and exclusion criteria

For the family, a positive family history comprised of one members diagnosed with CAD. The inclusion criteria for families in this study aligned with the clinical diagnostic criteria of CAD, focusing on patients newly diagnosed with CAD. This ensures that the study population consisted of individuals who meet the specific medical criteria for CAD diagnosis. The exclusion criteria served to exclude families who may have perplexing factors or conditions that could distress the study results or pose additional risks like tumour diseases, liver or kidney diseases, individuals undergoing other therapies and tested positive COVID-19 and those who may not comply with or cooperate with the treatment and study requirements (refused to sign consent form by any reason). By carefully selecting study participants based on these criteria, it was aimed to ensure that the study results were reliable, and any observed effects can be attributed to the treatment being investigated.

### Consent to participate and publication

Informed written consent was taken from all participants enrolled in the current study. All patients provided informed consent letter (written statement) who were 18 years old or above. Additionally, patients with less than 18 years of age, the written consent form was taken from legal guardians of patients. Consent letters were signed by patients/ or their guardian (below aged 18) to ensure patient confidentiality and legality in families and also have their contacts number to identify the individuals/patients during and after the data collection for further information in future if we needed. The Performa was intended to collect data on gender, age group, age of diagnosis, family history, different risk factor (high blood pressure, diabetes, cholesterol, creatinine level, cancer, and kidney failure), sedentary lifestyle (diet, smoking, stress, area of living, physical activity). BMI was calculated based on the height (m) and weight (kg) of everyone, according to WHO criteria (BMI = kg/m^2^). Body mass index was determined from the participant’s self-reported weight and height.

### Blood sample collection

**Fig 1:**
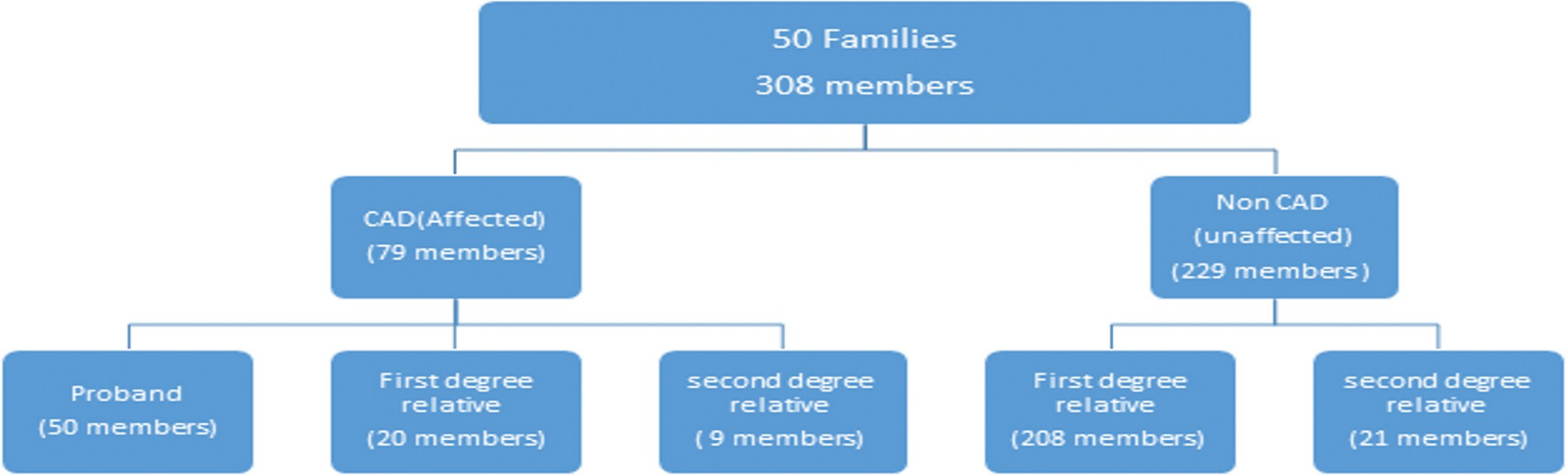
Flow sheet diagram of specifies of sampling.

Blood samples were collected from families (who were willing to become research participants) between September 2020 to August 2021. 50 families (mostly were nuclear families) in which overall 79(50 Proband(Patient), 20 FDR’s and 9 SDR’s) were diagnosed with CAD (Affected) and 229 (208 FDR’s and 21 SDR’s) were Non CAD (Unaffected) who participated in the research. In 50 families, 50 Proband were present, first degree relatives included Parents, siblings and children who shared approximately 50% of their genetic material with individual, second degree relatives included grandparents, grandchildren, aunts, uncles, nieces, nephews, and half-siblings. The orders of participants were shown in flowsheet diagram below Fig 1. 5 milli litre of venous blood was drawn from all the participants, which was collected in EDTA containing blood collection tubes at room temperature.

### DNA extraction

Genomic DNA was extracted by both using commercial kit for DNA isolation which named as Thermo Scientific™ GeneJET Genomic DNA Purification Kit and also used organic method [22] and stored at −20°C. The purity and concentration of genomic DNA was measured by Nanodrop (Thermo Scientific *NanoDrop*™ 3300 fluorospectrometer) at the wavelength of 260/280nm.

### Genes and SNP’s selection

Genes were selected based on their direct or indirect association with Selection of genes mostly depends on the already reported genes which were either directly or indirectly associated with the disease pathogenicity, level of cholesterol and inflammation from National Center for Biotechnology Information (NCBI; http://www.ncbi.nlm.nih.gov) and HapMap data bank. The selected different genes with their selected SNPs are enumerated in Table 1.

**Table 1:** Selected genes, snps, primers, product size and their restriction enzymes.

Genes	SNP	Primer	Product size	RFLP
*PCSK9*	Rs505151	Forward 5’GATGTCGGAGGGAGAAATGA‘3 Reverse 5’GGCACCCAGAGTGAGTGAGT‘3	287,215	Sau96I
*LDLR*	rs1122608	Forward 5’GAACGCCCCTCAAGCTGCCCTCC‘3 Reverse 5’AGCCACCGTGCCCAGCCTCCAA‘3	419	BsrI
*LDLR 2*	rs2228671	Forward 5’CTCTCAGTGGGCGACAGACG‘3 Reverse 5’CAACATGGCGAGACCCTGTC‘3	194	BstUI
*LPA-PLAG2*	rs1051931	Forward 5’TTTGTCCTGAGATTCATCTGGTT‘3 Reverse 5’ACTGGCAAAATAATTGGACACA‘3	159	AvaII
*LPA-PLAG2*	rs1805017	Forward 5’ACAGAGGTATTTGAGTCCCCAC‘3 Reverse 5’AATGTTGCCCATAAGCCAGT‘3	231	MnII
*TNF-ALPHA*	rs1800629	Forward 5’AGGCAATAGGTTTTGAGGGCCAT‘3 Reverse 5’CATCAAGGATACCCCTCACACTC‘3	107	NcoI
*ENOS*	rs2070744	Forward 5’GCAGGTCAGCAGAGAGACTA‘3 Reverse 5’GACACAGAACTACAAACCCC‘3	178	MspI
*ENOS*	rs1799983	Forward 5’GTCCCTGAGGAGGGCATGAG‘3 Reverse 5’TCCAGCAGCATGTTGGACAC	371	BanII
*APOE*	rs429358, rs7412	Forward 5’TCCAAGGAGCTGCAGGCGGCGCA‘3 Reverse 5’GCCCCGGCCTGGTACACTGCCA‘3	218	AflIII, HaeII
*MEF2A*	rs325400	Forward 5’CAGCCCCGACAGGAAATGG‘3 Reverse 5’GGAGAATGGAAGTCGCCCC‘3	385	HhaI
*PON1*	rs 662	Forward: 5′-TATTGTTGCTGTGGGACCTGAG-3′ Reverse: 5′-CACGCTAAACCCAAATACATCTC-3′.	99	AlwI

### Primers designing

The primers that were previously reported were selected and validated by retrieving sequences from the NCBI website. Their specificity was confirmed using the NCBI human genome database with BLAST (http://www.ncbi.nlm.nih.gov/BLAST/). The forward and reverse primers were selected for amplifying the targeted DNA fragments are listed in Table 1.

### Polymerase chain reaction (PCR) amplification

The already reported primers used in the reaction were as followed given below in Table 1. Each APOE, ~200 ng of the DNA template has been taken for PCR reaction, and Takara kit PCR mixture contained 2.5ul (10× PCR buffer contain MgCl2, 2ul of the reported reverse and forward primers each, and 2 microliters of 10mM of each deoxynucleotide triphosphate (dNTP); 2ul of Taq polymerase (TAKARA) and 1ul of 5% DMSO in a PCR reaction tube. PCR was carried out with following steps: 95°C for 3 minutes, 40 cycles of melting at 95°C for 60s, annealing at 58°C for 60s, extension at 72°C for 90s. The process established with a final elongation at 72°C for 10 minutes. After PCR, the final products were run on a 2% agarose gel for electrophoresis. The resultant 218bp product was then stored at 4 degrees Celsius for further digestion and analysis.

For LDLR rs1122608 and rs 22228671, about ~200 ng of the DNA template has been taken for PCR reaction, and Takara kit PCR mixture contained 2.5ul (10× PCR buffer contain MgCl2, 2ul of the reported reverse and forward primers each, and 2ul of dNTP, 2ul of Taq polymerase (TAKARA) and 1ul of 5% DMSO in a PCR reaction tube. PCR amplification was were carried out through following steps: First step is Denaturation at 95 degrees Celsius for 5 min, followed by 35 cycles of Melting for 1 min at 95°C, at 58°C for 1 min, at 72°C for 1 min, Final step for 7 min at 72°C and for rs2228671, Denaturation at 94 degrees Celsius for 3 min, 35 cycles = 1. 30s at 94°C/1min at 58°C/1 min at 72°C, Final step at 72 degrees Celsius for 7 min respectively. The resulting products of 419bp and 194bp were then stored at 4°C respectively.

Each LPA-PLAG2 rs1051931, rs1805017, reaction mixture was contained 100 ng DNA and KOD DNA Polymerase kit PCR mixture contained 2.5ul (10× PCR buffer, 1ul of 25mM MgCl2, 0.5ul of the reported reverse and forward primers each and 2.5ul of (dNTP); 0.8ul of Taq polymerase (KOD) and 1ul of 5% DMSO in a tube. PCR amplification steps were as followed, 3 min at 95°C for denaturation, reaction followed by 30 cycles 30s at 95 degrees Celsius, for 45 s at 58°C, for 1 min at 72°C, at 72°C for 7min. The 159bp amplicons of rs1051931 and 231bp products were of rs1805017 stored at 4°C.

For rs505151 mixture contained ~100 ng DNA template, and KOD DNA Polymerase kit PCR mixture contained 2.5ul (10× PCR buffer, 1ul of 25mM MgCl2, 0.5ulof the reported reverse and forward primers each, and 2.5ul of (dNTP); 1ul of Taq polymerase (KOD) in a PCR tube. PCR reaction steps were followed by, 3 min at 94 degrees Celsius, 30 cycles were used at 94°C for 30s/45 s at 55°C/30 s at 72°C. 5 minutes at 72° degrees Celsius for the elongation was used. The 194bp template was stored for further in future use. For TNF-ALPHA 100 ng of the DNA template, and KOD DNA Polymerase kit PCR mixture contained 2.5ul of PCR buffer, 1ul of 25mM MgCl2, 1ul of the reported reverse and forward primers each and 2ul of dNTP; 2ul of Taq KOD in a PCR reaction tube. 12 minutes at 94 degrees Celsius, 35 cycles were used at 94°C for 30 seconds/60s at 60°C/2 minutes at 72°C. 7 minutes at 72° degrees Celsius for the elongation was used. The 107bp template was stored for further in future use.

PON1 100 ng of the DNA template, and KOD DNA Polymerase kit mixture contained 2.5ul of PCR buffer, 1ul of 25mM MgCl2, 0.5ul of the reported reverse and forward primers each and 2.5ul of (dNTP); 1ul of Taq KOD in a PCR tube. PCR reaction was carries out by following steps: 5 minutes at 95 degrees Celsius, 35 cycles were used at 95°C for 60 seconds/1 minute at 61°C/1 minute at 72°C. 10 minutes at 72° degrees Celsius for the elongation was used. The 99bp template was stored for further in future use.

Each ENOS rs1799983 and rs2070744, 100 ng of the DNA template, and KOD DNA Polymerase kit PCR mixture contained 2.5ul of PCR buffer, 1ul of 25mM MgCl2, 0.5ul of the reported reverse and forward primers each and 2ul of dNTP, 0.8ul of Taq KOD in a PCR tube. PCR reaction was carries out by following steps: 4 minutes at 95 degrees Celsius, 35 cycles were used at 95°C for 30 seconds/1 minute at 61°C/half minute at 72°C. 7 minutes at 72° degrees Celsius for the elongation was used. The 178bp amplicons of rs1051931 and 371bp template of rs1805017were then stored at 4°C until analysis and digestion done.

For MEF2A, 100 ng of the DNA template, and KOD DNA Polymerase kit PCR mixture contained 2.5ul of PCR buffer,1ul of 25mM MgCl2, 0.5ul of 2.0 mM of each of the reported forward and reverse primers, and 2ul of (dNTP); 1ul of Taq KOD polymerase in a PCR tube. 3 minutes at 95°Celsius was used for denaturation, 35 cycles of 30 seconds at 95°C, 1 minute at 57°C, 30 seconds at 72°C, at 72°C for elongation 10 min were used. The resulting 385bp products were then put in storage at 4°C for forthcoming use.

### Genotyping (RFLP & Sanger Sequencing)

#### RFLP

Recipe

**Table:** 

10ul	PCR product
1ul	Restriction enzyme
2ul	Buffer
7ul	water

ApoE genotyping was conducted by digestion of the PCR product with AflIII and HaeII restriction (molecular scissors) enzymes in the presence of 10X buffer and BSA 0.2 μl. The mixture was then incubated at 37 degrees Celsius for 24 hours. The products after digestion were separated on a 2% gel(agarose) and the fragments of DNA were examined and documented by GelDoc photography. The PCR products of LDLR rs1122608, rs 2228671 digested at 37°C overnight and 6–10 hours in a volume of 25ul using BsrI and BstUI. The products were examined and separated on the 3.5%. gel(agarose). Genotyping for LPA-PLAG2 was accomplished by digestion of the PCR product with AvaII and MnII respectively and incubated for 24 hours at 37°Celsius. The resulting yields examined through 3% agarose (gel) electrophoresis. The PCR fragments were digested with 2 U of Sau96I for all night long at 37°C. The digestive yields were separated on 8% agarose gel. NcoI, AlwI, HhaI restriction endonuclease enzymes were used for the recognition of the TNF-alpha gene rs18000629 and PON1 rs662, MEF2A rs325400 for all night long at 37°C respectively. The resulting products were examined on 3% agarose gel electrophoresis. PCR products of eNOS rs1799983, rs 2070744 digested at 37°C overnight and 6–10 hours in a volume of 25ul using BanII and MspI. The resulting yields were examined on 3% agarose gel electrophoresis. Genotyping of PCR products were bidirectionally confirmed by Sanger sequencing (BGI Genomics, Hong Kong). Both methods are used to investigate the association in case-control study as well as association in families and their transmission throughout the families

### Statistical analysis

The data values were expressed as mean ± SD, numbers and percentages. The association between different independent and categorical variables was evaluated using the χ2 square test. The data analysis was done by using Graph Pad PRISM 9.0, with significance value p < 0.05. Pedigrees for each family were generated using PROGENY software (https://pedigree.progenygenetics.com/). The Pearson correlation between CAD and various risk factors/ complications was analysed in the Origin Tab 9.0. For families of coronary heart disease, the Transmission Disequilibrium Test (TDT) was used to evaluate association of family based testing for disease. Family-based association tests for disease traits (DFAM) and quantitative traits (QFAM) were conducted using PLINK dos-1.07 (Linux). The sanger sequences have been already submitted in the GENEBANK of NCBI. Linkage analysis was done by MERLIN software. The position of chromosomes were determined by MareyMap online program https://lbbe-shiny.univ-lyon1.fr/MareyMapOnline/.

## Results

### Demographic statistical results

**Fig 2:**
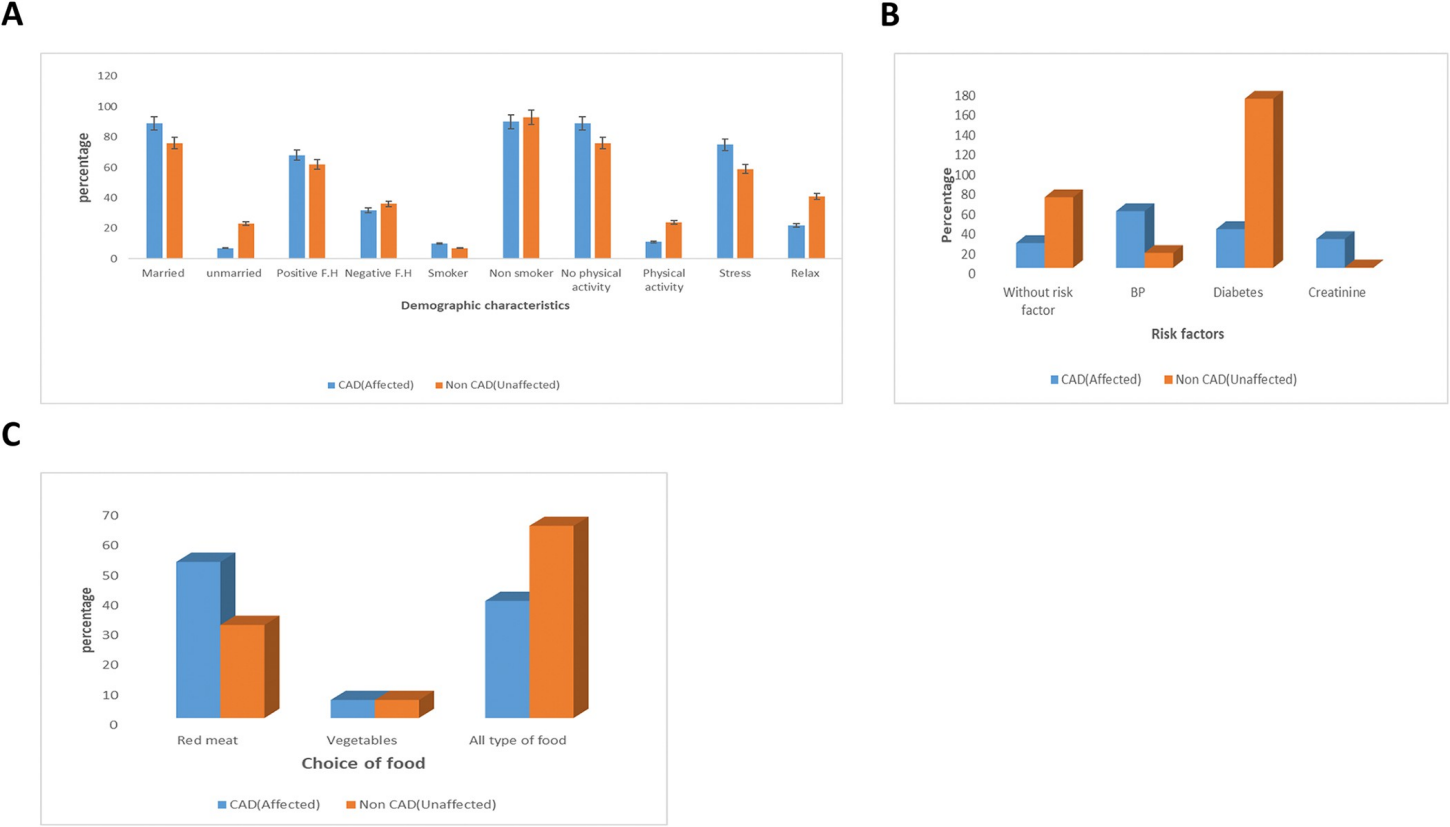
Evaluation of different demographic characteristics between affected and unaffected within families. (A) Distribution of Demographic characteristics among affected and unaffected persons among families, (B) Distribution of Risk factors associated with CAD among affected and unaffected persons among families(C) Distribution of food among affected and unaffected persons among families.

Fifty families were included in the study with total 308 participants; 79 members were affected with CAD and 229 were unaffected. The demographic and clinical were investigated for affected and unaffected families (Table 2). Males were 53 and females were 26 in affected persons and in unaffected males were 118 and female were 111. The significance level in chi square χ2 square was P < 0.05. The mean±SD values of age in affected and unaffected were 50.32±13.42 and 36.29± 14.75 respectively. The age of diagnosis value in patients in families was 47±13.49. The value of BMI in patients were 25.1±4.09 and in healthy persons was 22.1±5.09. The significance level in χ2 is p<0.05. The mean and SD value of Cholesterol, LDL and HDL was 242.7±47.9, 168.75±33.4 and 52.6±16.0 (Table 2). In cases, 10% were smoker, 90% were nonsmoker and in healthy 93% smokers and 7% were nonsmoker (Fig 2A). The ratio of stressed people was 75% and 22% were relaxed. Blood pressure 56% in patients is the one of the foremost cause in the prognosis of CAD. Diabetes in patients was 39%, creatinine level was 29% (Fig 2B). Red meat 52% is one of the leading cause of coronary artery disease 6% have prior to eat vegetables and 39% have showed all type of food (red meat, white meat, vegetables and pulses (Fig 2C).

**Table 2:** Demographic characteristics of affected (CAD Patients) and Unaffected (non CAD).

No.	Characteristics	Affected (CAD patients)	Unaffected (Non CAD)	P value (chi square χ2)
1	Gender a) Male b) Female	53 26	118 111	P<0.05
2	Age	50.32±13.42	36.29±14.75	P<0.05
3	Age of diagnosis	47±13.49	-----	
4	BMI	25.1±4.09	22.1±5,09	P<0.05
5	Family history a) Positive F.H b) Negative F.H	0.68 0.31	0.62 0.36	P<0.05
6	Risk factors a) No risk factor b) BP c) Diabetes d) creatinine	20(25.3) 45(56.9) 31(39.2) 23(29.1)	162(70.7) 22(9.6) 19(8.2) 1	P<0.05
7	Cholesterol	242.7±47.9	-------	
8	LDL	168.5±33.4	-------	
9	HDL	52.6±16.0	-------	

### Phenotypic pedigree analysis

The pedigree pattern of the families demonstrated the CAD transmission from Proband to their first-degree relatives(FDR) and Second degree relatives (SDR). There is no data for third degree relatives (TDR). The phenotypic pedigrees indicate the disease status and other associated risk factors and sedentary lifestyle. The research suggested that FDRs exhibit a higher susceptibility to CAD compared to SDRs because they share 50% of their genetic material with the individual. The family pedigrees(phenotypic) included in the study were provided as a supplementary data (S1–S5 Figs).

### Correlation between CAD and other risk factor/ complications

**Fig 3:**
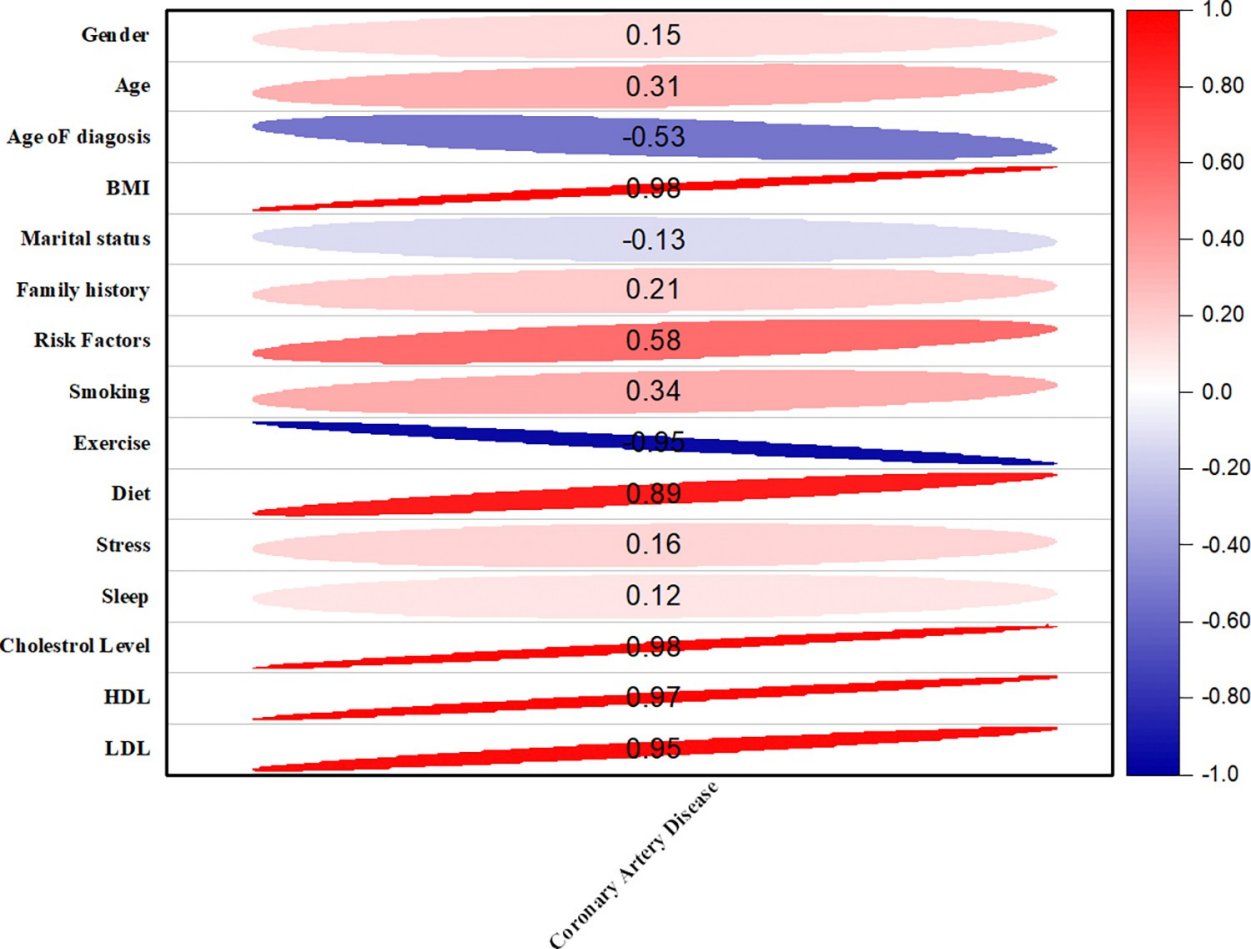
Graphical representation of correlation between CAD and various risk factors/complications. Closer to 1 in red showed positive correlation, closer to 0 showed non- significant, value toward -1 were found to be negative correlated.

Various risk factors/ complications had been correlated in the development of CAD. The positive correlation showed between gender and CAD (r = 0.15). Age and CAD showed positive correlation (r = 0.31). The age of diagnosis and CAD were also found to have a negative correlation (r = -0.53). Marital status showed a negative correlation with CAD (r = -0.13). The CAD and BMI were strongly correlated (r = 0.98). Family history and CAD had positive correlation (r = -0.21). CAD and the risk factors were also found strong positive correlation (r = -0.58). Stress and sleep with CAD showed favorable correlation (r = 0.16, r = 0.12) respectively. There was a found unfavorable relationship between CAD and exercise (r = -0.95). Diet and CAD were found to have positive correlation (p = 0.89). Cholesterol and CAD had strong positive correlation (p = 0.98). The level of HDL and LDL also have positive correlation with CAD (r = 0.97, r = 0.95) respectively as shown in (Fig 3).

### RFLP

**Fig 4:**
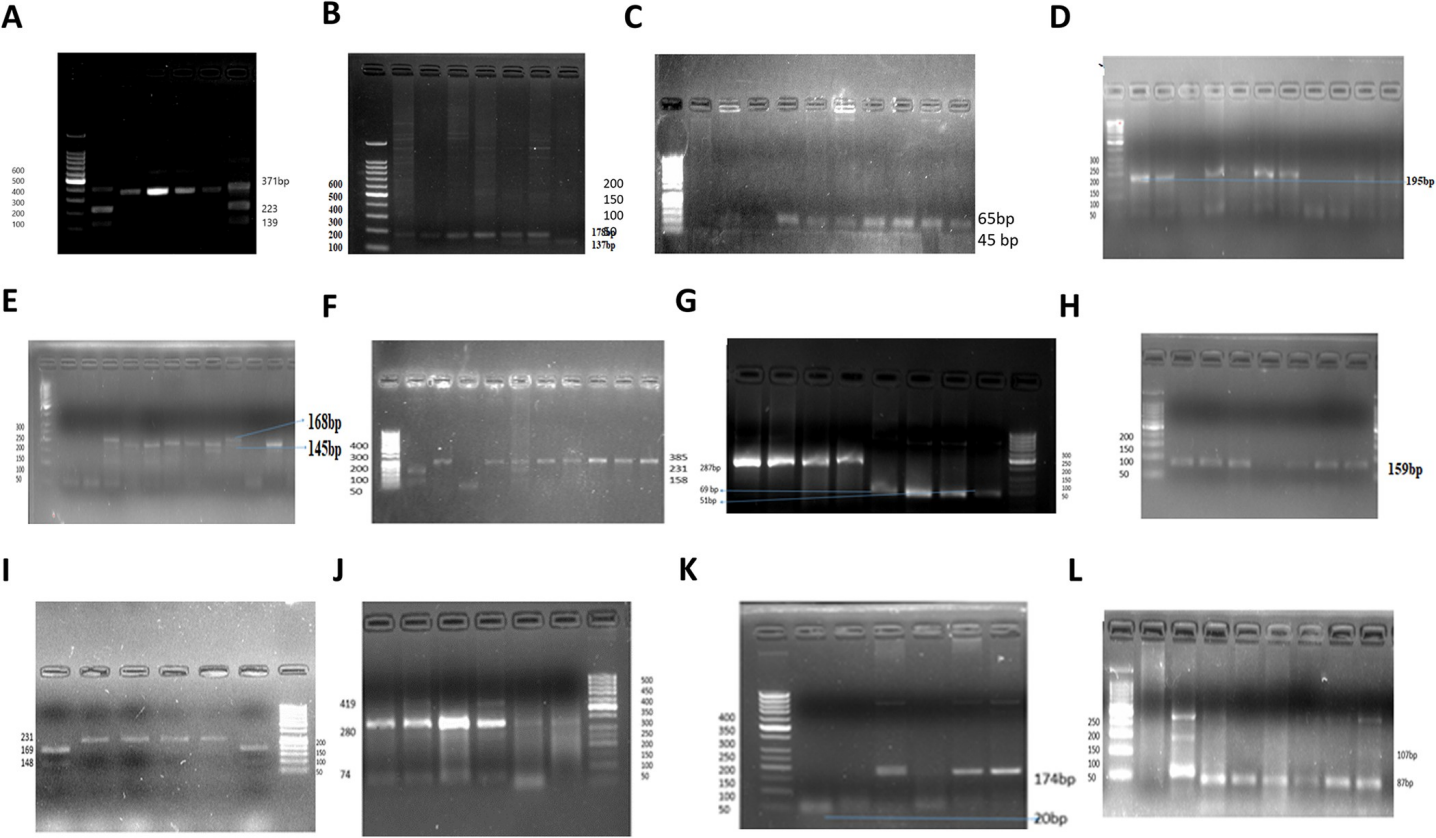
Representation of PCR-RFLP gel electrophoresis showing genotypes. (A) Representation photograph of RFLP of NOS3 gene rs1799983 showed 371bp, 223bp and 139 band size, (B) rs2070744 showed 178bp and 137bp band size, (C) PON1 gene rs662 showed 99bp, 65bp and 45bp band size. (D) APOE gene showed 168bp for E2, 145bp for E3 digested by HaeII, (E) 195bp for E4 by AflIII, (F) MEF2A gene rs325400 showed 385bp, 231bp and 158bp band size, (G) PCSK9 gene rs505151 showed 287bp, 69bp and 51bp band size, (H) LPA-PLA2 gene rs1051931 showed 159bp band size showed no restriction cut, (I) rs1805017 showed 231bp, 169bp and 148 bp band size, (J) LDLR gene rs1122608 showed 419bp, 280bp and 74bp band size and (K) rs2228671 showed 174bp, 20bp band size, (L) TNF- alpha gene rs180069 showed 107bp, 87bp.

The presence and sizes of bands correspond to the specific alleles detected after restriction digestion illustrated as (Fig 4A–4L). This was a genotyping representation of RFLP results of *PCSK9*, *PON1*, *LDLR*, *NOS3*, *LPA-PLA2*, *TNF*, *MEF2A*, *APOE*.

### Transmission disequilibrium test (TDT)

The paternal and maternal association test is truly relying on counting the no. of alleles in unaffected and affected parents. In TDT NOS3 (rs1799983), PON1 (rs662), LPA-PLA2 (rs1805017), MEF2A (rs325400), and LDLR (rs1122608, rs222867) showed association and transmission within families p≤ 0.05 whereas NOS3 (rs2070744), APOE (rs429358, rs7412) and TNF (rs1800629) showed no association TDT asymptotic (p<0.05) as shown in Table 3.

**Table 3:** Transmission disequilibrium test between families of CAD.

Gene	SNP	A1	A2	T	U	OR	L95-U95	P	P_PAR	P_COM
PON1	662	T	C	2	9	0.22	0.44–1.02	**0.03**	0.01	0.003
NOS3	1799983	G	T	0	4	0	0	**0.04**	1412.061e-005	8.029e-006
NOS3	2070744	C	T	2	3	0	0	0.1	0.04	0.2
TNF	1800629	A	G	1	4	0.25	0.02–2.23	0.1	0.03	0.3
LPA-PLAG2	1805017	C	T	2	1	0.3000	0.08–1.09	**0.05**	0.05	0.1336
MEF2A	325400	G	T	0	5	0.1	0.03–0.16	**0.02**	0.07	0.04
LDLR	1122608	C	T	2	9	0	0	**0.02**	0.007	0.04
	2228671	G	T	0	4	0	0	**0.04**	0.07	0.0006
APOE	429358	T	C	2	3	0.66	0.11–3.99	0.6	1	0.705
	7412	T	C	7	3	2.3	0.6–9.0	0.2	0.01	0.03

A1: Minor allele, A2: Major allele code, T: Transmitted minor allele count, U: Untransmitted allele count, OR: TDT odds ratio, P: TDT asymptotic p-value, P_PAR: Parental discordance asymptotic p-value, P_COM: Combined test asymptotic p-value.

### DFAM: Association test for disease traits in families

In DFAM test, NOS3 (rs1799983), PON1 (rs662), MEF2A (rs325400), and LDLR (rs1122608, rs222867) showed that sibling of Proband were at high risk of transmission p≤ 0.05 whereas NOS3 (rs2070744), APOE (rs429358, rs7412), LPA-PLA2 (rs1805017) and TNF (rs1800629) showed low risk of transmission asymptotic p-value >0.05 (Table 4).

**Table 4:** DFAM analysis test between families of CAD.

No.	Chromosome no.	SNP	A1/A2	OBS/EXP	CHISQ	P value
1	6	rs1800629	A/G	11/12.83	1.704	0.19
2	6	rs1805017	C/T	8/8.333	0.05556	0.8137
3	7	rs662	T/C	3/8	3.88	**0.02**
4	7	rs1799983	G/T	6/9	3.6	**0.05**
5	7	rs2070744	C/T	10/11.3	1.32	0.25
6	15	rs325400	G/T	2/6	3.85	**0.04**
7	19	rs1122608	C/T	1/3.5	3.90	**0.04**
8	19	rs2228671	C/T	11/9	6	**0.01**
9	19	rs429358	T/C	19/6.833	1.397	0.2373
10	19	rs7412	T/C	16/15	0.2609	0.6095

A1:A2: Minor and major allele codes, OBS: Number of observed minor alleles, EXP: Number of expected minor alleles, CHISQ: Chi-squared test statistic, P: Asymptotic p-value.

### QFAM: Association test for quantitative traits in families

In QFAM test, NOS3 (rs1799983, rs2070744), PON1 (rs662), MEF2A (rs325400), APOE(rs7412), LPA-PLAG2(rs1805017) and LDLR (rs1122608, rs222867) showed association p≤ 0.05 whereas APOE (rs429358) and TNF (rs1800629) showed no association EMP1 p-value > 0.05 as shown in Table 5.

**Table 5:** QFAM test analysis between families of CAD.

No.	Chromosome no.	SNP	STAT	EMP1
1	6	rs1800629	0.4153	0.4564
2	6	rs1805017	3.228	**0.05**
3	7	rs662	6.469	**0.01**
4	7	Rs1799983	44.41	**1e-005**
5	7	rs2070744	5.244	**0.03**
6	15	rs325400	17.27	**1e-005**
7	19	rs1122608	17.27	**3e-005**
8	19	rs2228671	3.69	**0.05**
9	19	rs429358	0.5951	0.3968
10	19	rs7412	4.759	**0.03**

CHR: Chromosome code, SNP: SNP identifier, STAT: Test statistic, EMP1: Pointwise empirical p-value.

### Linkage analysis

**Fig 5:**
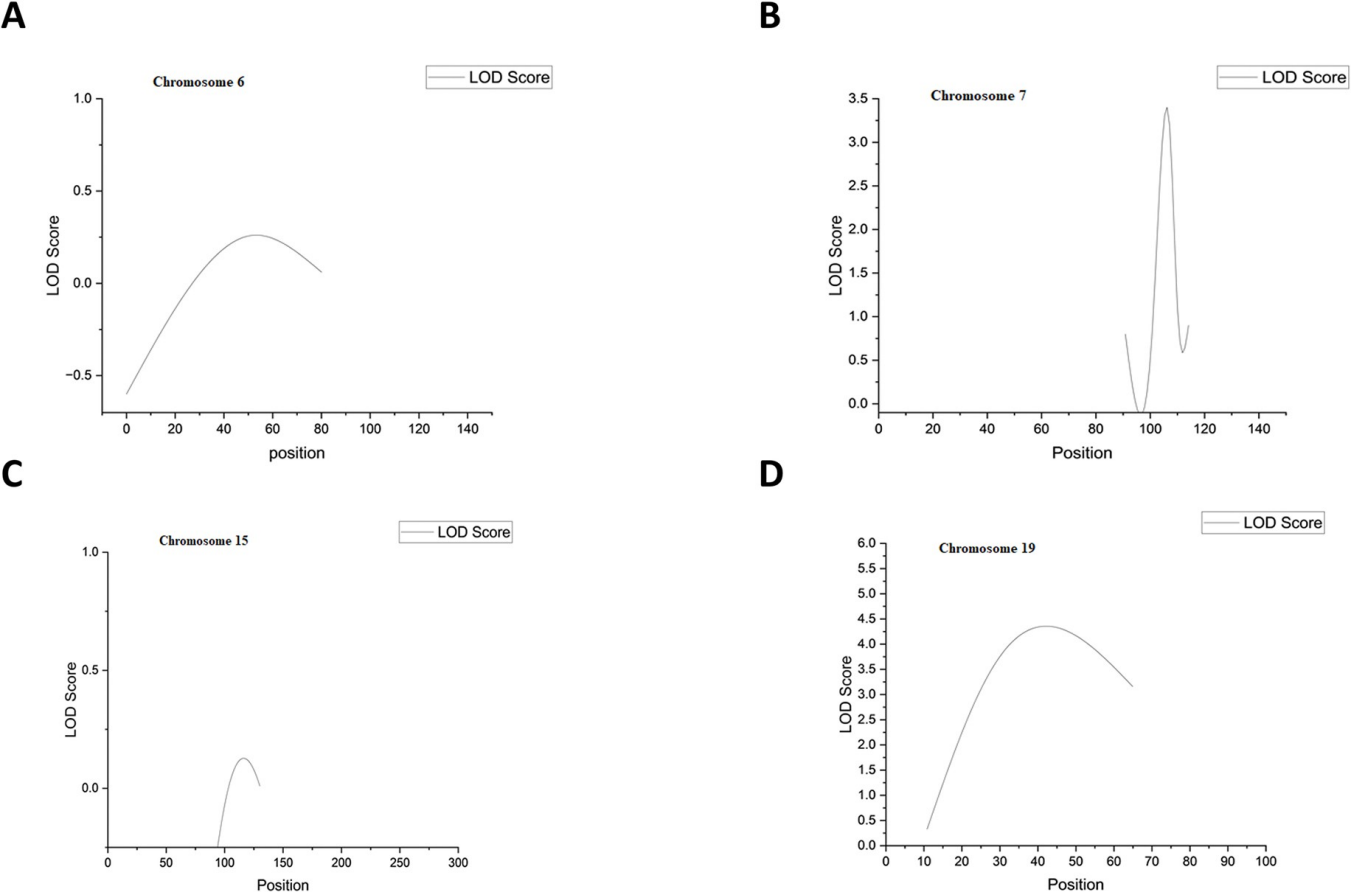
Graphical representation of Linkage analysis in families. (A) Graphical representation of LOD score on chromosome 6, (B) graphical representation of LOD score on chromosome 7, (C) graphical representation of LOD score on chromosome 15, d: graphical representation of LOD score on chromosome 19.

The results included chromosome number, Z-scores, p-values, LOD scores, and their associated p-values across specific positions. On the Chromosome 6 (Position 70.810): **LOD = 3.16**, p < 0.05 showed the significant evidence for linkage. Chromosome 7 (Position 107.190): **LOD = 3.16**, p < 0.05 and chromosome 19 (Position 31.470): **LOD = 3.90**, p < 0.05 also showed significant association for linkage with disease. Chromosome1(Position 22.700): **LOD = 0.53**, p> 0.05, chromosome 6(position = 51.710, 70.800, 70,810) and on the chromosome no 7 (position = 169.830, 169,840) showed no linkage with the CAD as p > 0.05 (Table 6). The graphical representation of threshold peak also showed in the (Fig 5A–5D).

**Table 6:** Linkage analysis between chromosomal position and CAD.

Chromosome	Marker	Position(Cm)	Zmean	pvalue (Zmean)	LOD	pvalue (LOD)
1	505151	22.700	0.73	1.0	0.53	0.3
6	1800629	51.710	0.59	0.30	0.26	0.14
6	1051931	70.800	1.32	0.5	0.8	0.3
6	1805017	70.810	1.52	0.5	0.8	**0.05**
7	662	107.190	3.20	**0.01**	**3.16**	**0.01**
7	2070744	169.830	0.12	0.50	0.80	0.30
7	1799983	169.840	0.12	0.50	0.80	0.70
15	325400	128.790	0.35	0.60	0.05	0.70
19	2228671	31.400	2.23	**0.03**	2.08	**0.02**
19	1122608	31.470	3.75	**0.04**	**3.90**	**0.02**
19	429358	64.930	3.10	**0.02**	2.16	**0.04**
19	7412	64.931	3.20	**0.02**	**3.16**	**0.02**

**Position** = region under study, **Z Mean** = Average Z score, **LOD** = Logarithm of odds, where values ≥3 are significant for genetic linkage. A p-value < 0.05 is typically considered significant.

## Discussion

The prevalence of coronary heart disease is widely increasing in Pakistan due to an ethnicity, lack of knowledge, and a poor lifestyle. CAD remains an intricate disease increasing day by day and influenced by genetic as well as by environment. Owing to its complication, the exact underlying grounds are unremarkably not clear. CAD cannot be cured but treatment can help to succeed in the diagnosis of symptoms early and reduce the risks of problems such as heart attacks in future. CAD could have caused by inheritance of genes but there’s no particular gene that causes heart disease alone, numerous genes can work together to surge the chances the progression and prognosis of CAD. Certain genes can also cause different risk factors such as high BP or cholesterol which become the main reason in developing of CAD.

Therefore, the present study emphasizes familial accretion of CAD and highlights the implication of detecting risk factors within the families in Pakistan. However, as the present study was consisted of 50 families (Proband, FDR and SDR) and so, the results from our research put through its paces on a large scale family so that outcomes can be used to change in lifestyle of people of Pakistan and to identify the predisposed persons in families and CAD early onset can be prevented or delayed by taking preventive measures with a better lifestyle.

The present study put emphasis on the demographic characteristics such as age, BMI, level of cholesterol, smoking and choice of their food. Diabetes mellitus, high BP, tobacco use, high lipid profile, obesity and stress are among the risk factors associated with Coronary Artery Disease (CAD). Extensive research and trials have led to the development of effective strategies for managing and preventing CAD [2]. Many individuals in the general population exhibit risk factors for coronary artery disease (CAD), including high blood pressure, lipid and lipoprotein metabolism disorders, diabetes mellitus, chronic kidney disease, age, gender, lifestyle choices, smoking, poor diet, obesity, and a family history of the condition [3]. Modifiable risk factors (high cholesterol levels, smoking, blood pressure, BMI, diabetes, and a poor diet, physical idleness) and non-modifiable factors included age, sex, ethnicity and family history were recognizing factors involved in the progression of CAD [23]. Globally, high blood pressure, smoking, elevated blood glucose, physical inactivity, overweight, and obesity are the six leading risk factors for diseases. Modifiable or treatable factors such as high blood pressure, abnormal blood lipids, and elevated blood glucose significantly contribute to the global incidence and burden of disease [24]. For both men and women, older age, diabetes, and hypertension are key risk factors, with hypertension being particularly significant in men [25]. Patients with CAD are more likely to smoke, have dyslipidemia, be diabetic, have a body mass index (BMI) over 30 kg/m^2^, have a family history of early-onset CAD, and be male [26]. Tobacco smoking stands out as a major risk factor for atherosclerosis, profoundly affecting its pathophysiology [27].

We explicitly recognized the NOS3 TT genotype in 12 patients (11.11%) and didn’t track down similar genotype in any of the controls. The frequencies of T allele in patients and the controls were 24% and 17.8%, separately [28]. Our findings suggest a substantial association between the homozygous genotype RR in PON1(rs662) and a higher prevalence in the CAD group compared to the healthy group (OR = 1.965, 95% CI = 1.223–3.159, p = 0.005) [14]. This current study showed that no GA and GG found in our population. Likewise, a solid affiliation was noticed for computer aided design risk with OR = 1.590, 95% CI = 1.106–2.284 and P = 0.012. Be that as it may, the homozygous GG freak genotype was viewed as totally missing from our population [29]. The rs1122608 had showed the strong association with CAD in case control study and also in families. Our outcomes presented that genotypes of homozygote rs1122608 (P<0.0001), rs10417578 (p<0.007) and rs4300767 (P<0.005) SNPs together have shown protective effect. Moreover, rs1122608 was at greater risk in prognosis of CAD (P = 0.01) [30]. A measurably critical affiliation was distinguished amid rs1122608 and risk of CAD by large examination in both Asian and Caucasian subgroups. The G allele of rs1122608 was associated high triglyceride level with cholesterol level, LDL cholesterol and high-thickness lipoprotein cholesterol level [31]. Present study has shown that rs325400 of MEF2A gene is strongly related to CAD in case control study and in families so, there was a strong association between rs325400 and CAD (χ^2^ = 24.77, P < 0.001) [32]. PON1 (Q192R) had substantial influence on the menace of CAD (*P* = 0.001). The carriers who had allele 192R considerably greater threat of developing CAD than other Q192 carriers [8,33,34]. Ma et al. found that the ε3/ε4 genotype and ε4 allele frequency were notably higher in patients with CAD compared to control participants. Among control participants, carriers of the ε2 allele indicated considerably lower levels of LDL-C and cholesterol and triglycerides (TG) in comparison to ε3 or ε4 carriers [9]. It was found that the frequency of APOE4 carriers (3/4 and 4/4 genotypes) was significantly higher in the severe stenosis group (≥70%) compared to the mild stenosis group (<30%) (22.8% versus 13.01%). In Multiple regression analysis showed that APOE4 carriers had an OR ratio of 2.16 for developing ≥70% stenosis. In conclusion, the presence of the APOE4 allele represents a significant risk factor for developing severe coronary stenosis (>70%) among individuals of Pakistani descent [35]. The current study showed TNF alpha polymorphism with CAD but had not any association within families. According to our findings demonstrated an association between the TNF-α-308G/A polymorphism and CAD patients with ≥50% obstruction, suggesting the necessity for additional investigations into the role of the TNF-α-308G/A polymorphism in hypertension [1,36]. The TT genotype of rs1799983(NOS3) in patients were found to be associated with the higher Body mass index (p = 0.068) [37,38]. The T allele heterozygosity in rs2228671 are powerfully related with enlarged predisposition to CAD [39]. All the snps of different genes in families, the study is limited by a small sample size in family data in Pakistani population as there was very less data of second degree relative and no third degree relative data, which restricts its ability to represent the genetic variety on a broad-range population. Recruiting more second-degree and third degree relative’s data is a valuable approach for studies that aim to extend the understanding of hereditary conditions of CAD, balancing broader family perspectives with practical and ethical considerations. CAD is influenced by both genetic and lifestyle factors; unravelling these effects remains a challenge which hinders a complete understanding of progression and development of CAD. The present-day study emphasis on genetic factors fails to see the possible influences from environmental effects, which hinders a complete understanding of progression and development of CAD. Given multifactorial nature of CAD future research should investigate these interactions to environmental and genetic diversity to gain more understanding of susceptibility of CAD in families.

Identifying genetic loci linked to CAD provides insights into the biological pathways and molecular targets mechanisms driving the disease. Genetic loci can contribute to estimate an individual’s predisposition to the disease. Genetic loci discovery reveals novel drug target and tailored therapeutic strategies. Identifying population-specific loci may lead to more accurate risk prediction and management in underrepresented groups.

## Conclusion

Our research work showed that the genetic variants NOS3 (rs1799983), PON1 (rs662), LPA-PLA2 (rs1805017), APOE (rs429358, rs7412), MEF2A (rs325400), TNF (rs1800629), and LDLR (rs1122608, rs2228671) were found to be associated with CAD (p < 0.05). Family-based association analysis revealed that the SNPs in NOS3 (rs1799983), PON1 (rs662), LPA-PLA2 (rs1805017), MEF2A (rs325400), and LDLR (rs1122608, rs222867) showed transmission within families (p < 0.05) in relation to CAD-associated genes. These polymorphisms in familial study relevant to CAD provide a better understanding of hereditary role with the interaction of other factors. In future, this type of study needs to be conducted on a broader scale on diverse sub-population groups to approve the impact of numerous hereditary and environmental determinants on multifarious CAD incidences. This study will help to identify the genetic predisposition in early detection and monitoring the individual at risk. Furthermore, this type of approach is recommended to better comprehend the disease pathology, to target the therapeutic agents in the perspective of a personalized medicine approach, understanding the disease mechanism and can lead to development of novel drugs. Insights into familial genetic risks can inform community-based preventive measures for CAD. Understandings the familial genetic risks can enlighten community-based genetic counseling and preventive measures for CAD but require careful management of the associated challenges and implications.

## Supporting information

S1 Fig Phenotypic pedigree of Family 1. (TIF)

S2 Fig Phenotypic pedigree of Family 2. (TIF)

S3 Fig Phenotypic pedigree of Family 3. (TIF)

S4 Fig Phenotypic pedigree of Family 4. (TIF)

S5 Fig Phenotypic pedigree of Family 5. (TIF)
