# Supplementary figures and images for: Identifying genetic susceptibility loci associated with human coronary artery disease
Source: PLoS One. 2025 Jan 9;20(1):e0315460. doi: 10.1371/journal.pone.0315460 (PMC11717286; doi:10.1371/journal.pone.0315460)

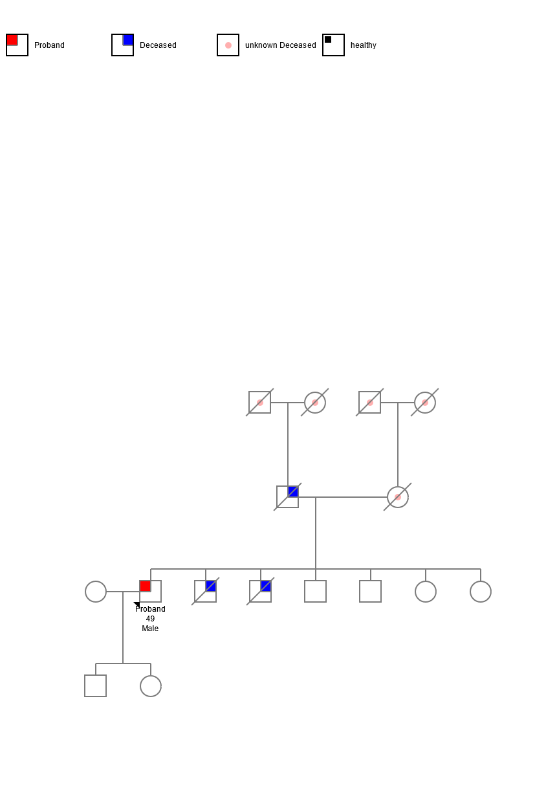

Supplement: S1 Fig — (TIF) [file pone.0315460.s001.tif]

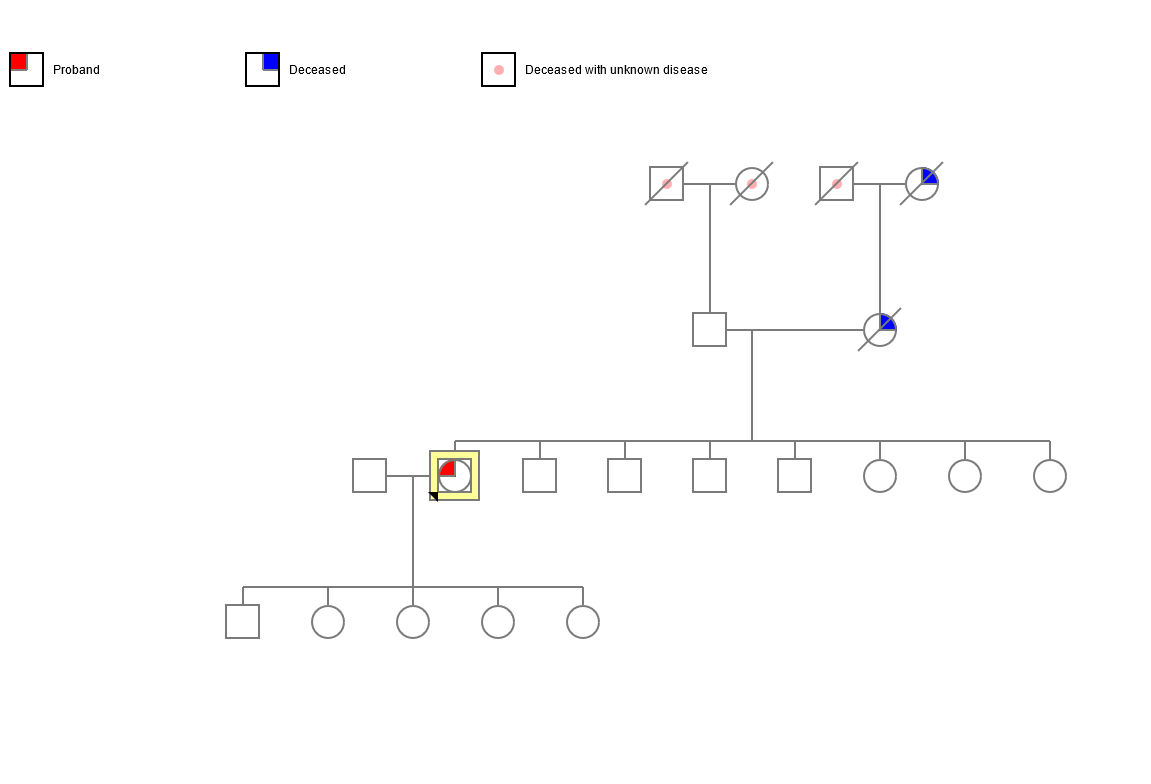

Supplement: S2 Fig — (TIF) [file pone.0315460.s002.tif]

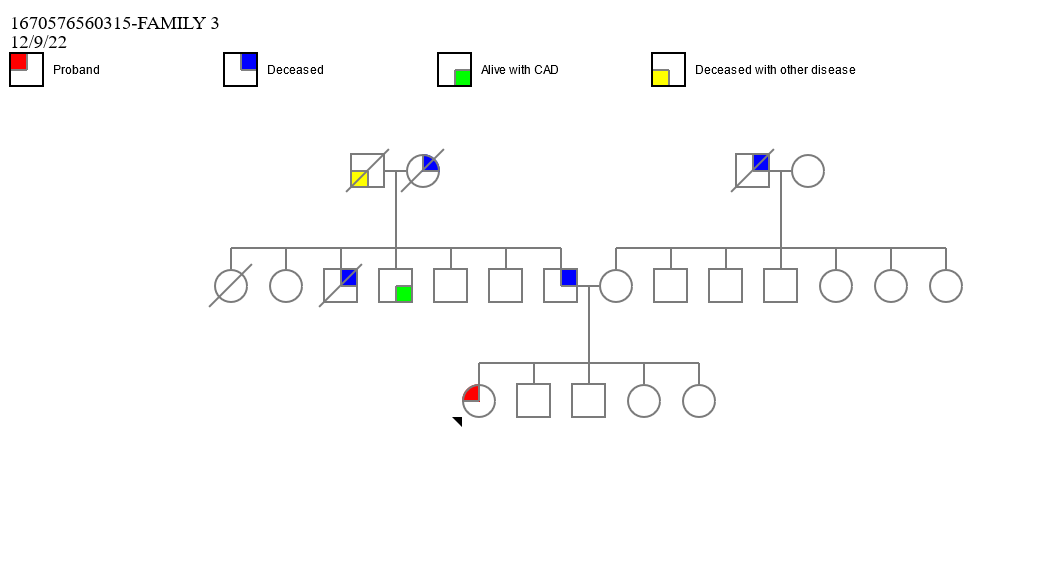

Supplement: S3 Fig — (TIF) [file pone.0315460.s003.tif]

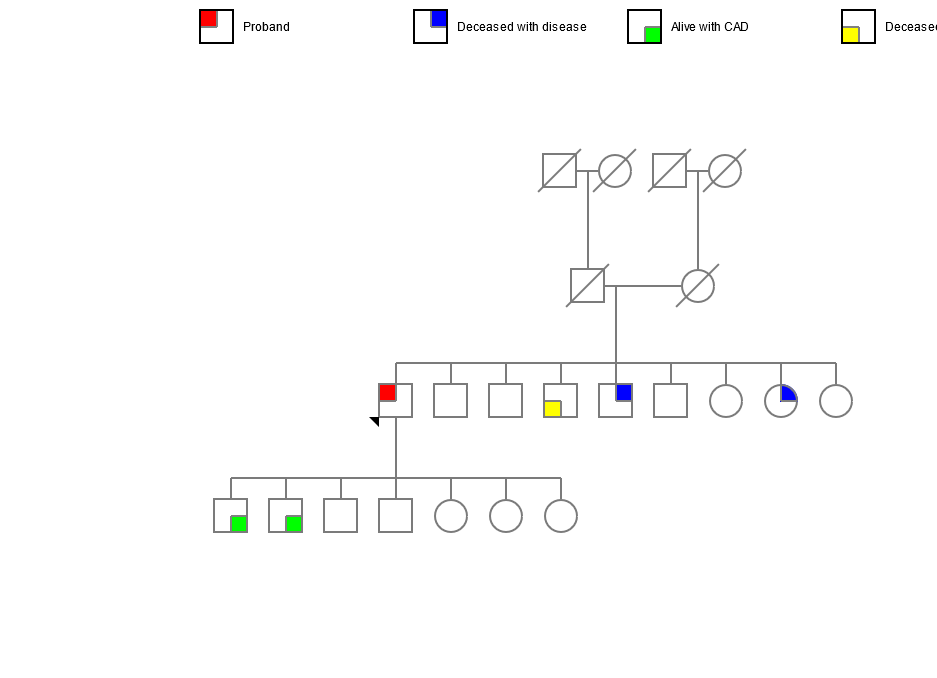

Supplement: S4 Fig — (TIF) [file pone.0315460.s004.tif]
